# The influence of water and air temperature on elite wheelchair triathlon performance

**DOI:** 10.1080/23328940.2024.2391170

**Published:** 2024-08-11

**Authors:** David N. Borg, Alexander D. Gibson, Aaron J.E. Bach, Emma M. Beckman, Sean M. Tweedy, Ian B Stewart

**Affiliations:** a School of Exercise and Nutrition Sciences, Queensland University of Technology, Brisbane, Australia; b Centre of Data Science, Queensland University of Technology, Brisbane, Australia; c School of Health Science and Social Work, Griffith University, Gold Coast, Australia; d Cities Research Institute, Griffith University, Gold Coast, Australia; e School of Human Movement and Nutrition Sciences, University of Queensland, Brisbane, Australia

**Keywords:** Athletic performances, heat stress disorder, para-athletes, sports for persons with disabilities, temperature

## Abstract

Impaired thermoregulatory function is a clinical feature of many health conditions that affect triathletes using wheelchairs and consequently, individual athlete performances may fluctuate according to environmental temperature. We aimed to determine the effect of 1) water temperature on wheelchair triathlon swim time and 2) air temperature on handcycle and wheelchair run (push) time. Published race records from 2017 to 2023 (*n* = 49 events) were extracted from the World Triathlon website. Bayesian negative binomial regression was used to separately model the nonlinear relationships between water temperature and swim time, and air temperature and handcycle and push time. Age, sex, sport class, whether wetsuits were worn (swim model), and swim time (handcycle and push model) were included as fixed effects. Over the observed water temperature range of 15.7–30.5°C, male swim time (mm:ss) improved from 14:13 (95% credible interval [CrI] = 12:27, 16:09) to 12:35 (95% CrI = 11:00, 14:19). Female swim time improved from 15:33 (95% CrI = 13:24, 17:55) to 12:46 (95% CrI = 11:03, 14:38). It was unclear whether handcycle and push time slowed over the observed air temperature range of 14–33°C. Warmer water temperatures, up to 30.5°C, were associated with faster swim times. It was unclear whether combined handcycle and push time slowed with increases in air temperature, up to 33°C. The integration of information on athlete impairment type and severity with performance data is needed to better understand the extent to which individual athlete performances fluctuate across environmental conditions.

## Introduction

Impaired thermoregulatory function is a clinical feature of many health conditions that affect triathletes who use wheelchairs, including spinal cord injury [1] and multiple sclerosis [2]. Yet, the impact of environmental heat on endurance exercise performance in Para athlete populations has received little scientific attention [3]. The only available study, a recent investigation by Alkemade et al. [4], compared time-to-exhaustion in the heat (31.9°C and 72% relative humidity [rh]) between elite Para (cycling and wheelchair tennis) and able-bodied athletes [4]. The study found that in hot-humid conditions, Para athletes experienced similar performance decrements to able-bodied athletes.

A study of Para triathletes competing in the 2017 World Cup (water 27°C; air 33°C; 41% rh) and 2018 World Series (water 25°C; air 33°C; 35% rh) reported that the core temperature of triathletes using wheelchairs increased by an average of 1.4°C (~0.018°C/min) from pre- to post-race [5]. While the magnitude of the core temperature change was likely confounded by athletes’ heat adaptation status, the study shows that triathletes using wheelchairs experience significant levels of thermal strain. For context, in able-bodied triathletes, the rate of change in core temperature over an Olympic distance event (air 19.3°C; 55% rh) has been reported to be 0.019°C per minute [6].

World Triathlon implements a heat policy where the swim segment is canceled when water temperatures exceed 32°C (section 17.11j in [7]), and events are rescheduled or canceled when wet bulb globe temperature (WBGT) exceeds 32.2°C (section 10.3 in [7]). The policy, however, was designed to protect athlete health [8], not declines in performance, which could still be significant even within these temperature limits [9,10]. With global temperatures set to increase, triathletes who use wheelchairs will be increasingly required to perform in challenging environments [11]. To best prepare athletes, studies on how the performances of triathletes using wheelchairs fluctuate with environmental temperature are needed.

Using World Triathlon data, we aimed to determine the effect of 1) water temperature on swim time, and 2) air temperature on combined handcycle and run (herein push) time, in triathletes who use wheelchairs. We hypothesized that 1) swim time would improve in warmer water, reach a plateau, and subsequently slow in hotter temperatures, and 2) handcycle and push time would slow in a nonlinear fashion with increases in air temperature.

## Materials and methods

### Study overview

Race data from World Championship Series, World Championships, World Para Cup, World Para Series events between 2017 and 2023, and the Tokyo Paralympics were retrieved from the World Triathlon website (https://triathlon.org/paratriathlon/results), on May 29 2023. Race results included performance times and, in most instances, water and air temperature. We retrieved data from 2017 onwards, as this period coincides with the current classification system [12]. Data were de-identified by removing athlete names. Ethical approval for the study was not required because race records are made publicly available.

### Wheelchair triathlon

Wheelchair triathlon is a multidisciplinary endurance sport that typically consists of a 750 m swim, a 20 km recumbent handcycle, and a 5 km run (push) in a racing chair, each separated by a brief transition. The swim is commenced in-water. Athletes are allowed to wear wetsuit bottoms, irrespective of the water temperature. Athletes may have one handler to support them during the two transition periods. Under the current classification system there are two sport classes, Wheelchair Triathlon Class 1 and Class 2 (section 17.2b in [7]). Class 1 athletes have more severe impairments than Class 2 athletes. Both sport classes compete in the same medal event. Male Class 1 athletes receive a time advantage of 03:00 min and female Class 1 athletes receive an advantage of 03:38 min (section 17.5i in [7]). On the elite circuit, Para triathletes compete in annual events scheduled in any season across different continents.

### Data and variables

Published separately for males and females, race records included a unique identifier for each athlete, a unique identifier for each race, the event location and year, swim, cycle, and run segment times, transition 1 and transition 2 time, and total race time. The athlete's year of birth was extracted from the World Triathlon website and used to calculate age. Race results are published with program notes, which generally include water and air temperature, and segment distances. Segment distance was used to calculate the average speed of the swim, cycle, and run for each athlete.

Air temperature was not defined; however, it was assumed to be dry bulb temperature, given that race conditions are monitored with a WBGT device (section 10.3 in [7]). Wet bulb globe temperature was not reported in the program notes, and no other information on the environmental conditions (e.g. relative humidity) was provided.

### Data analysis

Our primary interest was to investigate 1) the effect of water temperature on swim time and 2) the effect of air temperature on handcycle and push time.

Negative binomial regression was used to separately model swim time (in seconds) and combined handcycle and push time (in seconds). The relationship between water temperature and swim time was modeled using a natural cubic smoothing spline (knots at 22°C and 26°C), because we expected average swim times to improve as water temperature warms, plateaus, and subsequently slows in hotter temperatures. The location of the knots was chosen based on exploratory plots. The model included *sex* (levels: male, female), *age* (mean centered), *sport class* (levels: 1, 2), and *wetsuit* (levels: allowed, mandatory, not allowed) as fixed effects. A random intercept was included for each unique race, to account for differences in water conditions (e.g. currents) and race tactics [13]. The inclusion of year as a fixed effect could be used to account for policy and/or technology changes. However, there were no significant policy or technology changes between 2017 and 2023, so we opted to exclude year from the model, particularly because it was perfectly correlated with changes in age, which would have complicated the modeling approach [14].

Air temperature was included as a second-order polynomial (quadratic) term in the handcycle and push time model, because we expected handcycle and push time to slow in a nonlinear fashion with increases in air temperature. The model included *sex* (levels: male, female), *age* (mean centered), and *sport class* (levels: 1, 2), *swim time* (group [sex] mean centered [15]) and *swim time* by *sex* as fixed effects. Swim time was included to account for prior race performance, which could affect handcycle and run time. A random intercept was included for each unique race to account for differences in course geography and race tactics [13]. Year was not included for the reasons described above.

We considered the potential influence of event calendar on performance times, by including *month* as a random effect variable. Accounting for month had no impact on the substantive conclusions of the analysis (Supplement 1). Consequently, month was not included in the models to avoid overfitting [16].

Models were implemented in a Bayesian framework using Stan [17] with the *brms* interface [18]. A uniform prior distribution was used for the regression coefficients; a half *t*-distribution (df = 3, mean = 0, SD = 2.5) prior for the standard deviation of the random effects; and a Gamma (shape = 0.01, scale = 0.01) prior for the shape parameter. Posterior estimates were generated using Markov chain Monte Carlo methods (4 chains 10,000 iterations, 50% burnin, no thinning). Posterior estimates of interest were as follows: 1) the mean and 66% and 95% credible interval (CrI) of regression coefficients, *β*; 2) the probability that *β* was greater or less than zero, given by Pr *β* > 0 or Pr *β* < 0, depending on whether the mean effect was positive or negative; and 3) the estimated marginal mean (and 95% CrI) effect of water temperature of swim time, and air temperature on handcycle and push time. Posterior predictive checks were performed to assess the suitability of all models.

The number of events and athletes, proportion of females, and athlete age were summarized descriptively. Data cleaning, wrangling, and visualization were done using packages from the *tidyverse* [19]. Missing data were assumed to be missing at random [20]. Missing *x*-variable values (except for missing wetsuit data) were imputed using random forests [21], a nonlinear modeling technique that makes no assumptions about interactions in the data [22]. Missing wetsuit data were conditionally imputed based on the random forest imputed water temperature value, as follows: <18°C “mandatory,” 18°C to 24.5°C “allowed,” or >24.5°C “not allowed” (section 17.11f and 17.11h in [7]). The data and R code are available at https://doi.org/10.5281/zenodo.10774736.

## Results

### Data cleaning

A total of 639 records from 52 events between 2017 and 2023 were initially extracted. Three events (33 records from 6 races) were removed because the race was modified to a duathlon due to poor water conditions. Events from the following were included: Paratriathlon World Cup (*n* = 17), World Paratriathlon Series (*n* = 10), World Triathlon Para Cup (*n* = 9), World Triathlon Para Series (*n* = 7), World Triathlon Grand Final (*n* = 3), World Triathlon Championship Finals (*n* = 1), World Triathlon Para Championships (*n* = 1), and Paralympics (*n* = 1). Seven athletes did not start a race, 10 did not finish and 13 were disqualified. One data point was removed because it exceeded the maximum race limit of 2 h (see 17.5c in [7]), leaving 575 race records for analysis.

### Missing data

From 575 race records, missing data were as follows: air temperature 17%, water temperature 14.6%, wet suit information 12.9%, swim time 3.7%, push time 1.7%, handcycle time 1.6%, and sport class 0.2%. Supplement 2 shows the combinations of missing data.

### Paratriathlete demographics

**Figure 1.**
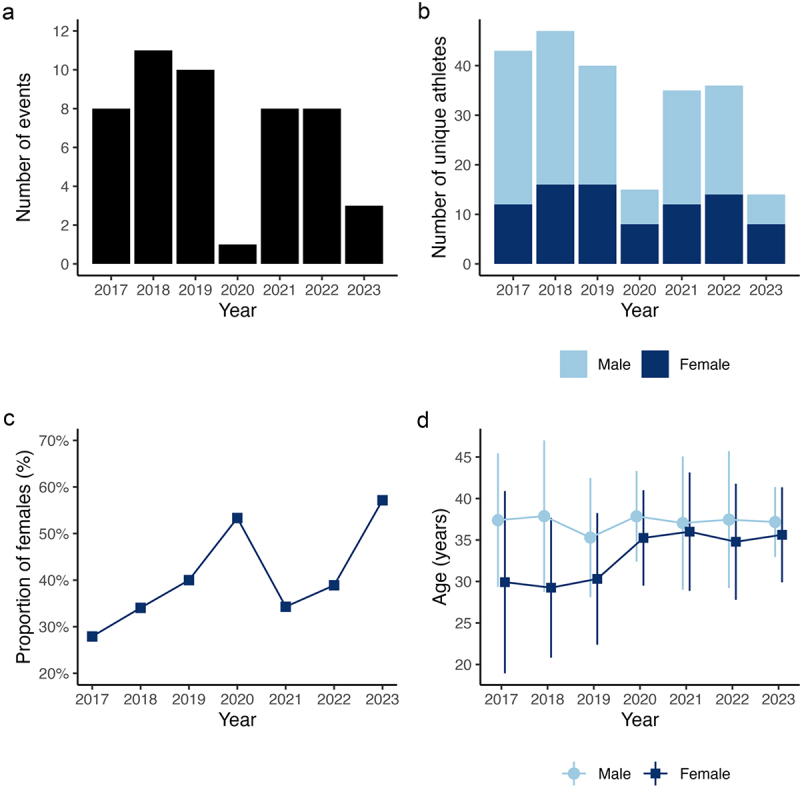
The number of wheelchair triathlon events (panel a), the number of unique male and female competitors (panel b), the proportion of female triathletes (panel c), and the age (mean with standard deviation) of male and female triathletes (panel d) between 2017 and 2023.

There were 79 unique triathletes (*n* = 25 females) in the dataset (Figure 1a). Figure 1b shows the proportion of female athletes between 2017 and 2023. The mean age of male athletes remained similar across the study period, at about 37 y (Figure 1c). The mean age of female athletes was 29.5 y in 2017, increasing by 5.7 y in 2023 (Figure 1c).

### Overall and race segment times

Table 1 provides a summary of segment and race performance time and speed for male and female athletes.

**Table 1.** Mean and standard deviation segment and overall race time and speed.

	Male Athletes		Female Athletes	
Segment	Time (min)	Speed (km·h^−1^)	Time (min)	Speed (km·h^−1^)
Swim	13.6 (2.9)	3.4 (0.5)	14.6 (2.3)	3.2 (0.5)
Handcycle	39.0 (6.3)	32.0 (4.4)	44.9 (6.9)	27.7 (3.6)
Run (push)	14.7 (3.0)	21.1 (3.0)	16.5 (2.6)	18.7 (2.6)
All^a^	71.1 (9.9)	NC	80.4 (9.0)	NC

NC = Not computed. ^a^ All includes transition 1 and transition 2 times.

### Water and air temperature

**Figure 2.**
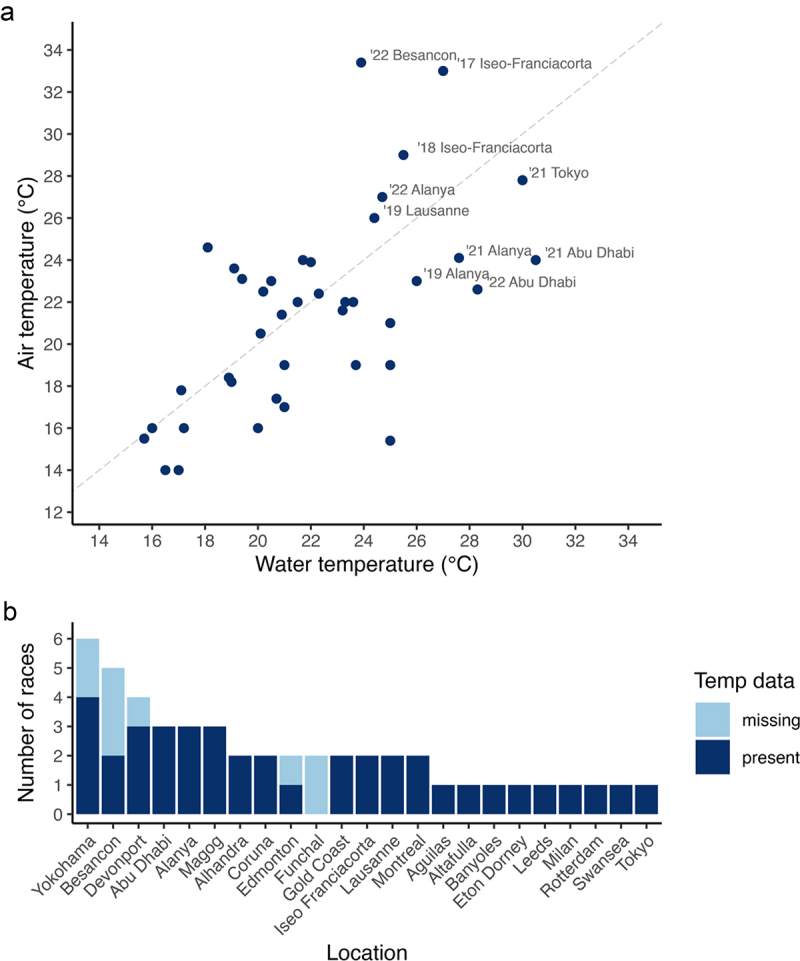
Water and air temperature from elite wheelchair triathlon events between 2017 and 2023 (panel a) and a summary of races (*n* = 49) according to location (panel b). In panel A, the dashed line represents the line of identity (*r* value of 1). Panel a location labels are provided for relatively hotter temperatures. Temp, temperature.

Figure 2a shows the relationship between water and air temperature. Water temperature ranged from 15.7°C to 30.5°C. Air temperature ranged from 14°C to 33°C. Figure 2b provides a summary of event locations, with missing temperature data indicated.

### Water temperature and swim time

**Figure 3.**
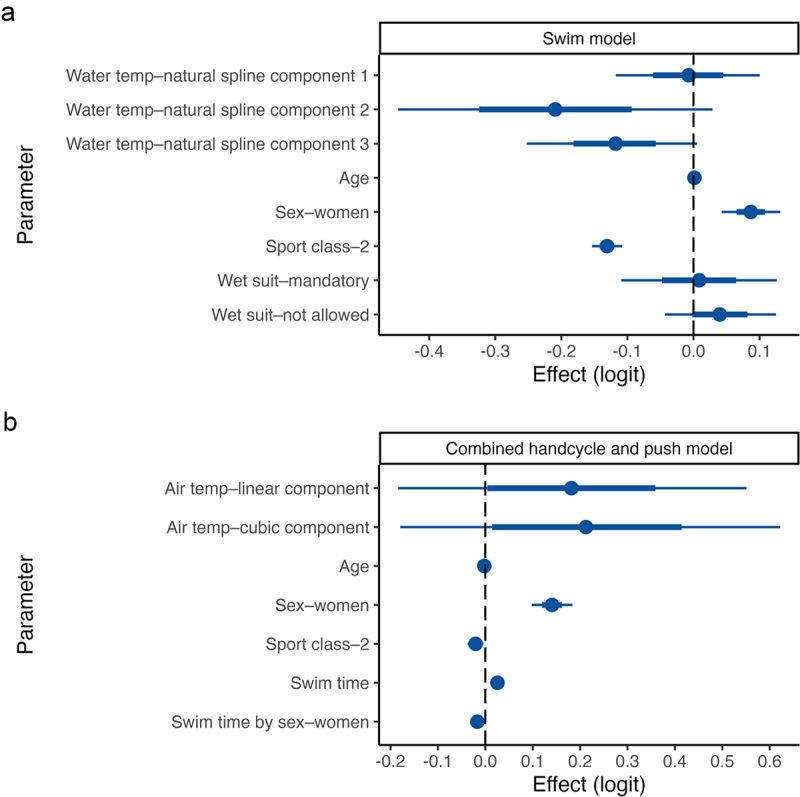
Posterior parameter estimates (logit scale) from the models of swim time (panel a), and handcycle and push time (panel b). The mean (circle) is shown with 66% (inner thick line) and 95% (outer thin line) credible intervals. Temp, temperature.

**Figure 4.**
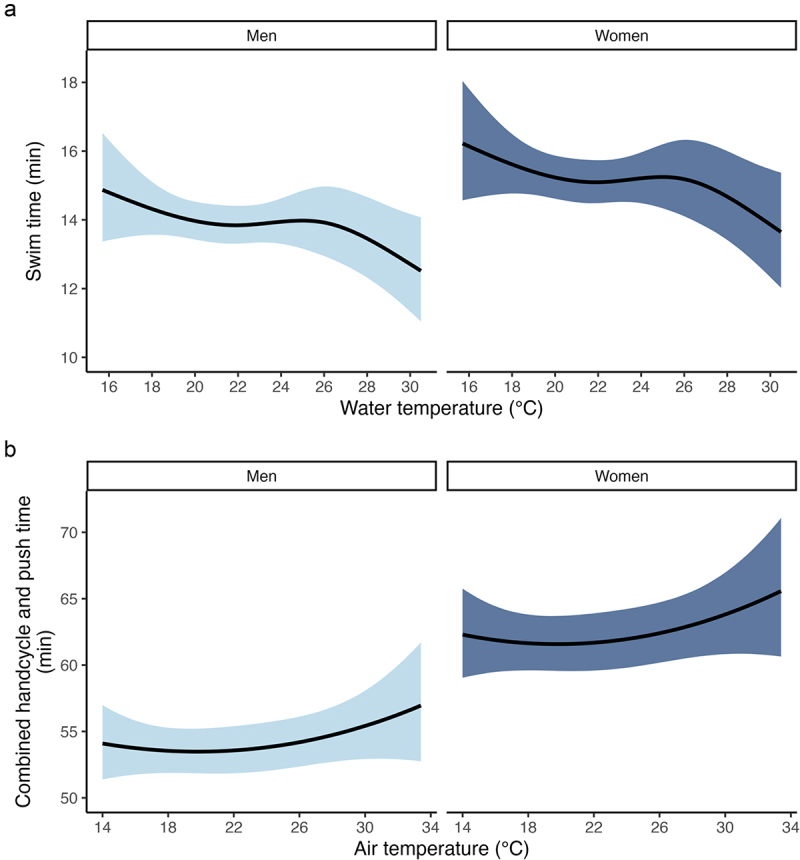
The relationship between water temperature and swim time (panel a) and air temperature and handcycle and push time (panel b). The solid black line indicates the marginal mean, and the ribbon indicates the 95% credible interval.

There was evidence that swim times decreased with increases in water temperature (Figure 3a and Figure 4a). There was a 95.8% chance that spline component 2 was less than zero (Pr *β* < 0 = .958), and a 97% chance that that spline component 3 was less than zero (Pr *β* < 0 = .97), see Figure 3a. Over the observed water temperature range of 15.7°C to 30.5°C, swim time (mm:ss) improved for men by 01:38 min, from 14:13 min (95% CrI = 12:27, 16:09) to 12:35 min (95% CrI = 11:00, 14:19), and improved for women by 02:47 min, from 15:33 min (95% CrI = 13:24, 17:55) to 12:46 (95% CrI = 11:03, 14:38). These changes represent a 11.5% change for men and a 17.9% change for women. A plot of individual athlete data is shown in Supplement 3 panel A.

There were also effects of sex (Pr *β* > 0 = 1), age (Pr *β* > 0 = .971), and sport class (Pr *β* < 0 = 1) on swim time (Figure 3a). Females swam 01:15 min (95% CrI = 00:35, 01:50) slower than males. For each 1-year increase in age, the swim time increased (slowed) by 0.49 s (95% CrI = 0.48, 0.50). Class 2 athletes swam 01:42 min (95% CrI = 01:28, 02:08) faster than Class 1 athletes. It was unclear whether there was a wetsuit effect on swim time – the posterior probability that *β*_mandatory_ and *β*_not allowed_ were greater than zero was 55.8% and 82% (Figure 3a).

### Air temperature and combined handcycle and push time

It was unclear whether handcycle and push time increased (slowed) with increases in air temperature (Figures 3b and 4b). There was a 83.6% chance that the linear component was greater than zero (Pr *β* > 0 = .836), and an 84.8% chance that the cubic component was greater than zero (Pr *β* > 0 = .848; Figure 3b). A plot of individual athlete data is shown in Supplement 3 panel B.

There were also effects of sex (Pr *β* > 0 = 1), age (Pr *β* < 0 = 1), and sport class (Pr *β* < 0 = .992) on swim time (Pr *β* > 0 = 1), and sex by swim time (Pr *β* < 0 = 1) on handcycle and push time (Figure 3b). Handcycle and push time was 08:06 min (95% CrI = 05:30, 10:27) slower for females than males. For each 1-year increase in age, handcycle and push time decreased (improved) by 13.95 s (95% CrI = 13.93, 13.96). Class 2 athletes were 01:05 min (95% CrI = 00:14, 02:08) faster than Class 1 athletes.

## Discussion

Our retrospective analysis of elite wheelchair triathlon competition data showed that warmer temperatures, up to 30.5°C, were associated with faster swim performances. It was unclear whether combined handcycle and push time slowed with increases in air temperature, up to 33°C. Athletes’ use of heat preparation strategies before races in warmer conditions, the presence of only mild heat stress, and/or convective cooling efficiencies may have contributed to the inconclusive result.

There was a marked difference in swim times between low (<18°C) and high (>26°C) water temperatures (Figure 4a). Faster performances in warmer water may be explained by an acute increase in physical function due to vasodilation and increases in muscle and tissue temperature. However, we could not find any evidence to support this statement. It is reasonable to assume that the warm-water benefit would be greatest for triathletes with health conditions where function is adversely affected by the cold, for example, cerebral palsy [23]. However, we found little evidence to suggest that sport class – in theory, a proxy of level of impairment – had a modifying effect on the relationship between water temperature and swim time (Supplement 4 panel A). In the interest of (not) overfitting the model [16], we did not include a temperature by sport class interaction.

As recommended [10,24], and based on previous studies [5,25], many athletes would have prepared for races in hot conditions by undertaking heat-based training. The use of heat preparation strategies along with more acute measures, such as pre-race ice-slurry ingestion, may have diminished the negative effect of heat on performance, leading to an unclear result [26,27]. The presence of only relatively mild heat stress could be another contributing factor. Water vapor pressure, a key component of environmental heat stress [28], was unknown. In active individuals, the risk of adverse heat effects begins to increase from WBGT 19–25.6°C [29]. These WBGTs could translate to a range of dry bulb temperature and relative humidity combinations. For example, assuming a solar load of 350 W/m^2^ and an airspeed of 5.5 m/s (20 km/h cycling with no wind), a WBGT of 24°C could translate to 24.5°C and 85%, or 35.4°C and 20%. Heat stress would be decreased in dry-warm conditions, due to the benefits of velocity-based (convective) cooling efficiencies during the handcycle and push segments [30,31]. Unfortunately, while World Triathlon uses a heat policy that relies on WBGT, these data were not reported in program notes.

There is a general lack of scientific literature concerning 1) the effect of heat on exercise and 2) the physiological risk of heat illness in the types of health conditions that affect triathletes using wheelchairs [3]. Evidence from indoor wheelchair rugby (air 18.4–20.9°C; 31–45% rh) indicates an inverse relationship between physical impairment and performance, such that athletes with severe physical impairments experience larger reductions in performance and greater thermal strain, due to their reduced heat loss capacity [32]. If the detrimental effect of heat on triathlon performance was associated with a level of impairment [32,33], it would stand to reason that Class 1 athletes (with the greatest impairments) may experience more pronounced heat-mediated declines in performance. However, we found little evidence to suggest that sport class had a modifying effect on the relationship between air temperature and performance (Supplement 4 panel B). We did not include an interaction between air temperature and sport class in our model, again to avoid overfitting [16].

### Limitations

World Triathlon program notes included water and air temperature but not WBGT data. As WBGT considers air temperature, relative humidity, and solar radiant heat, the relationship between WBGT and handcycle and push time could be different to the relationship between air temperature and handcycle and push time. We did not have any information on athlete type and impairment, which could have a modifying effect on the relationship between water and air temperature and performance [34–36]. Triathletes using wheelchairs can wear wetsuit bottoms irrespective of the water temperature. This could explain why the effect of wetsuit on swim times was unclear.

### Future directions

Future studies should integrate information on athlete impairment type and severity, with WBGT and performance data, to better understand how individual athlete performances fluctuate across environmental conditions. Such insights could be used to inform whether the time advantage received by Class 1 triathletes should vary as a function of the environmental conditions. While beyond the focus of the current study, we noted that the current World Triathlon heat policy used for Para athlete events is based on recommendations for non-disabled persons [8]. Future studies should explore whether a specific heat policy for Para triathletes is needed, tailored in a similar manner to the exertional heat stroke policy for Para athletes [37].

## Conclusion

Our analysis showed that in elite wheelchair triathlon events, warmer water temperatures were associated with faster swim performances. The effect of hotter air temperatures on handcycle and push time was inconclusive. There was little evidence to suggest that sport class had a modifying effect on the relationship between temperature and performance, in either the swim, or the handcycle and push. Athletes’ use of heat preparation strategies before races in warmer conditions, mild heat stress, and/or velocity-based (convective) cooling efficiencies may explain the inconclusive result. The integration of information on impairment type and severity, with WBGT and athlete performance data is needed to provide greater insight into how the performances of triathletes who use wheelchairs fluctuate across environmental conditions.

## Supplementary Material

Supplemental Material

## Data Availability

The data and R code are available at the doi:10.5281/zenodo.10774736.

## List of Abbreviations

CrI: Credible interval
df: Degrees of freedom
Pr: Probability
rh: Relative humidity
SD: Standard deviation
WBGT: Wet bulb globe temperature
