# Supplementary material for: The influence of water and air temperature on elite wheelchair triathlon performance
Source: Temperature (Austin). 2024 Aug 11;11(4):363–72. doi: 10.1080/23328940.2024.2391170 (PMC11583579; doi:10.1080/23328940.2024.2391170)

**Supplement 1.** Refitted performance time models, accounting for the potential influence of month. The analysis presented here explores whether environmental temperatures were related to the time of year. For example, were conditions cooler at preparatory races held earlier in the year, compared to major events in the European summer (mid-year). We first present exploratory plots of relevant variables, and second, show the parameter estimates and fitted values from refitted performance time models, with *month* included as a random effect variable.

The plot below shows the number of events in each month, by region. Male and female events are indicated by a different colour.


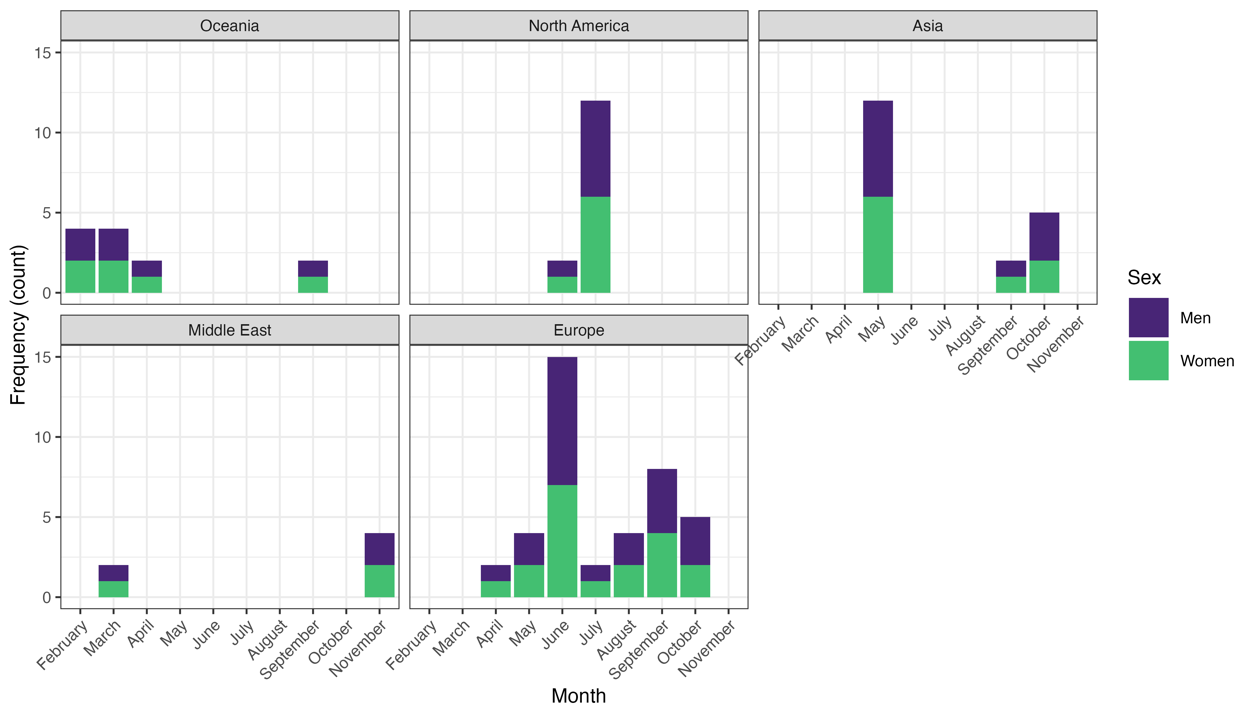


The plot below shows water temperatures for each month, faceted by year and sex. Colours indicate a different year.


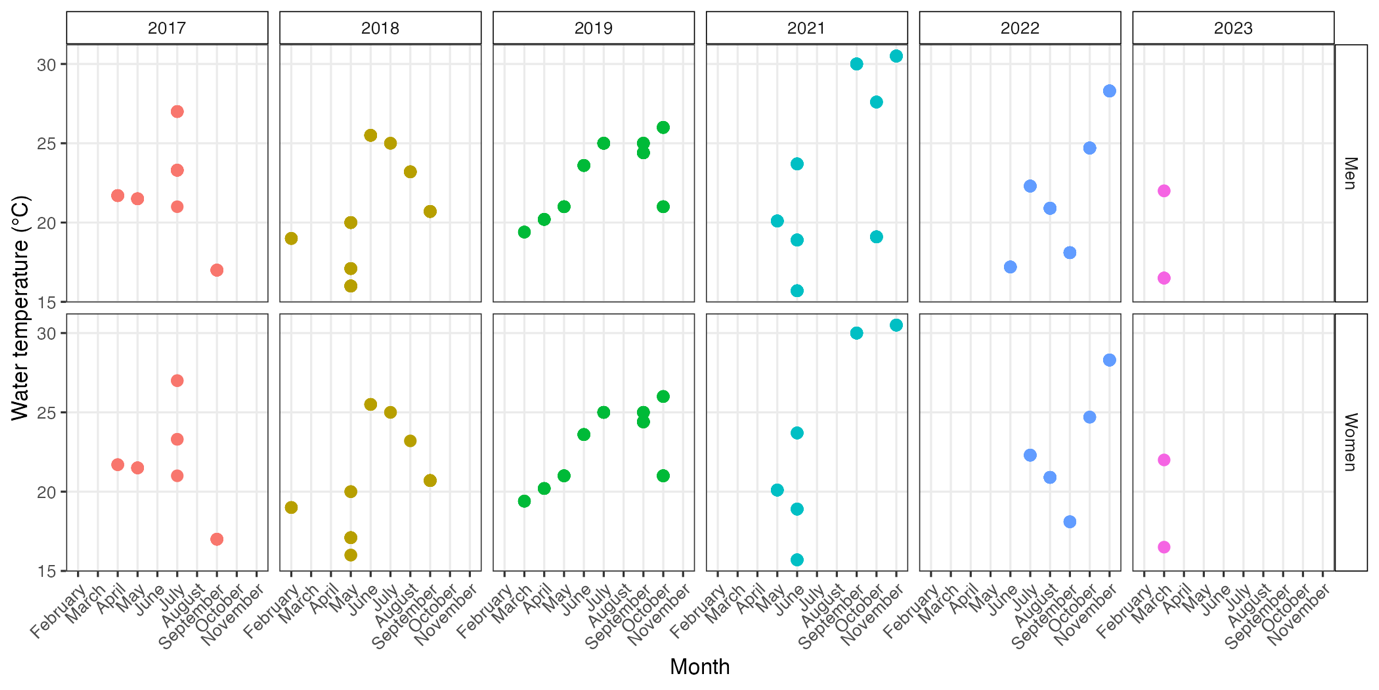


The plot below shows boxplots (and individual athlete data) of swim times for each month, separately for men and women.


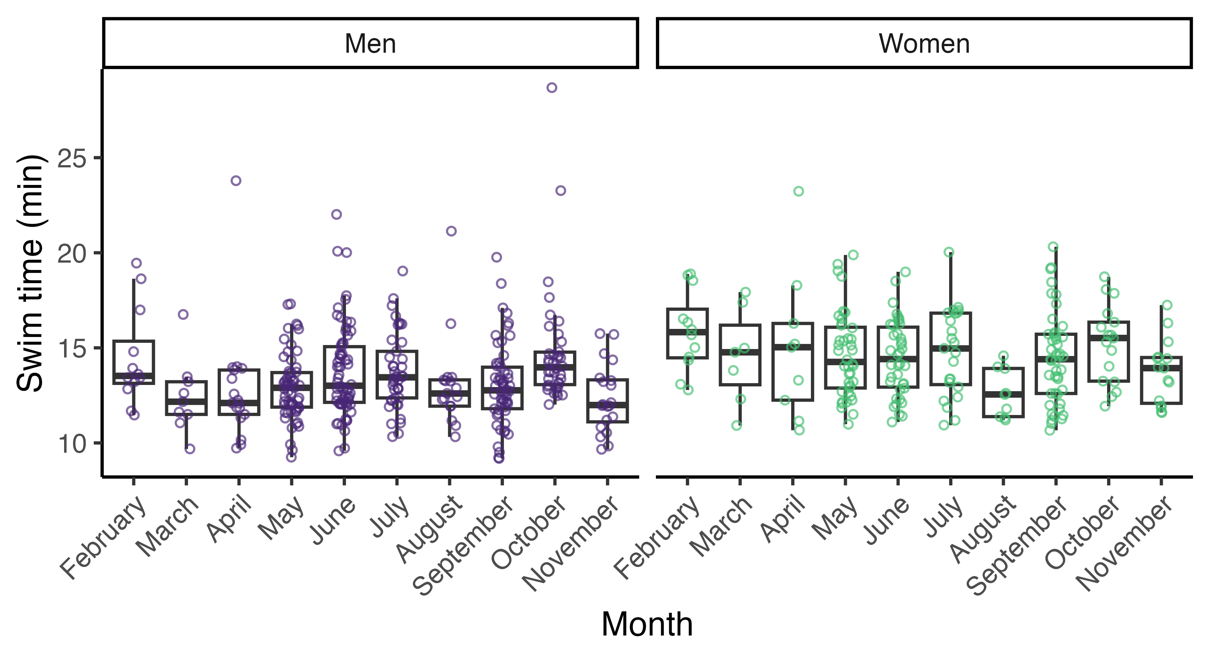


The plot below shows air temperatures for each month, faceted by year and sex. Colours indicate a different year.


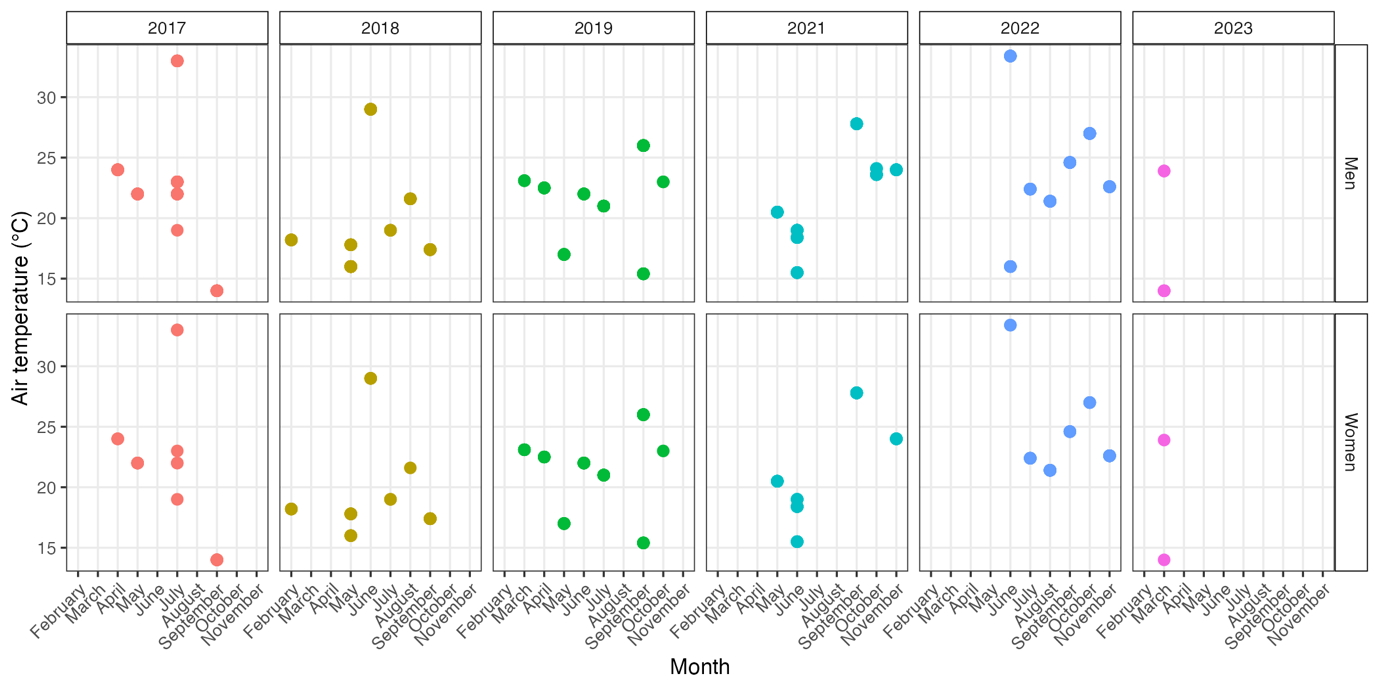


The plot below shows boxplots (and individual athlete data) of handcycle and push times for each month, separately for men and women.


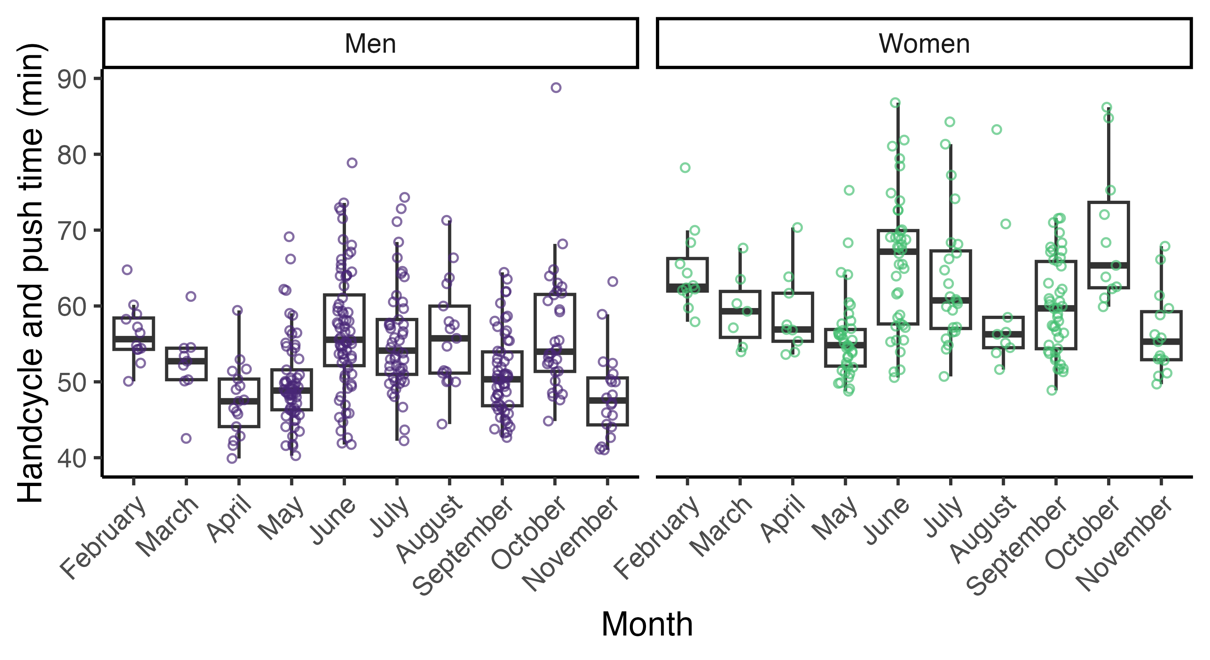


The plot below shows parameter estimates (on the logit scale) of fixed effects from the performance time models that include month as a random effect variable. The mean (circle) is shown with 66% (inner thick line) and 95% (outer thin line) credible intervals. Temp, temperature. Panel A shows parameter estimates from the swim model. Panel B shows parameter estimates from the handcycle and push time model.


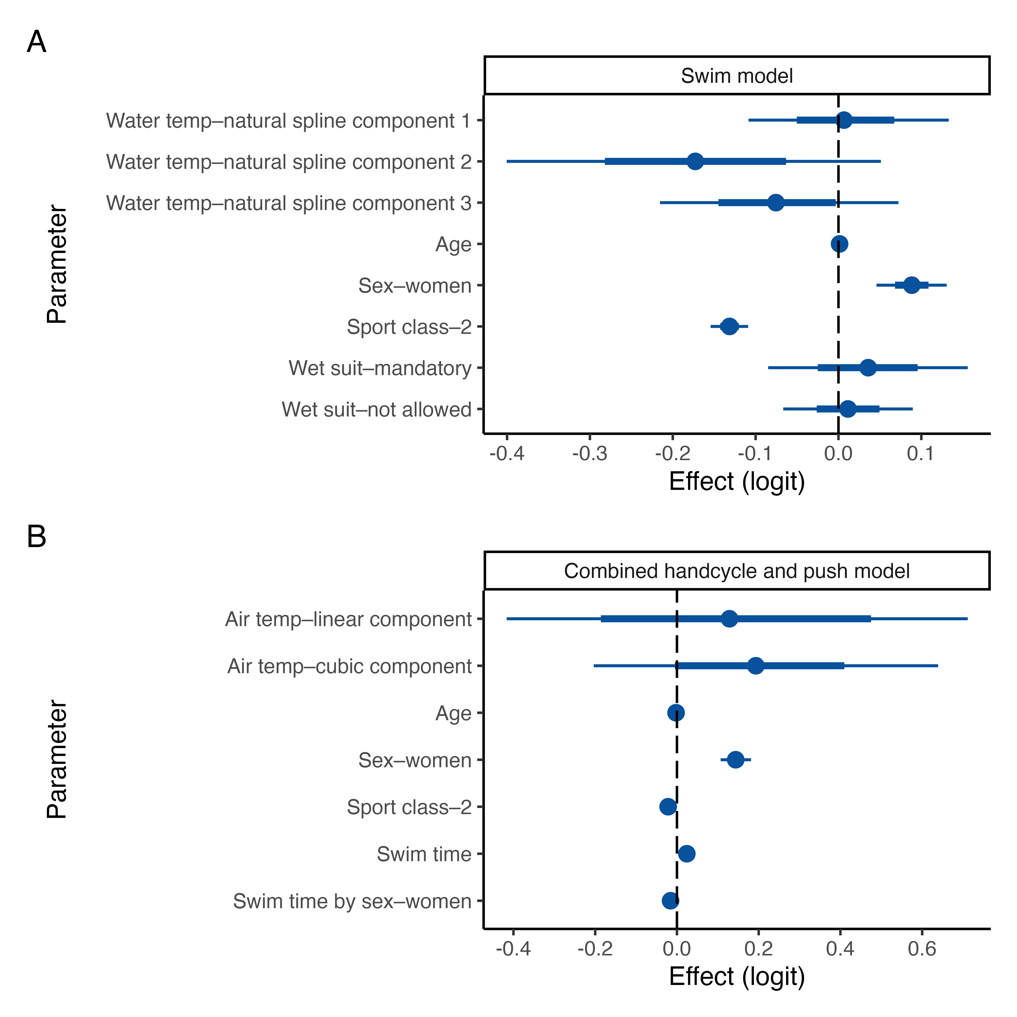


The plot below shows the fitted values from the performance models that include month as a random effect variable. Panel A shows the relationship between water temperature and swim time. Panel B shows the relationship between air temperature and handcycle and push time. The solid black line indicates the marginal mean, and the ribbon indicates the 95% credible interval.


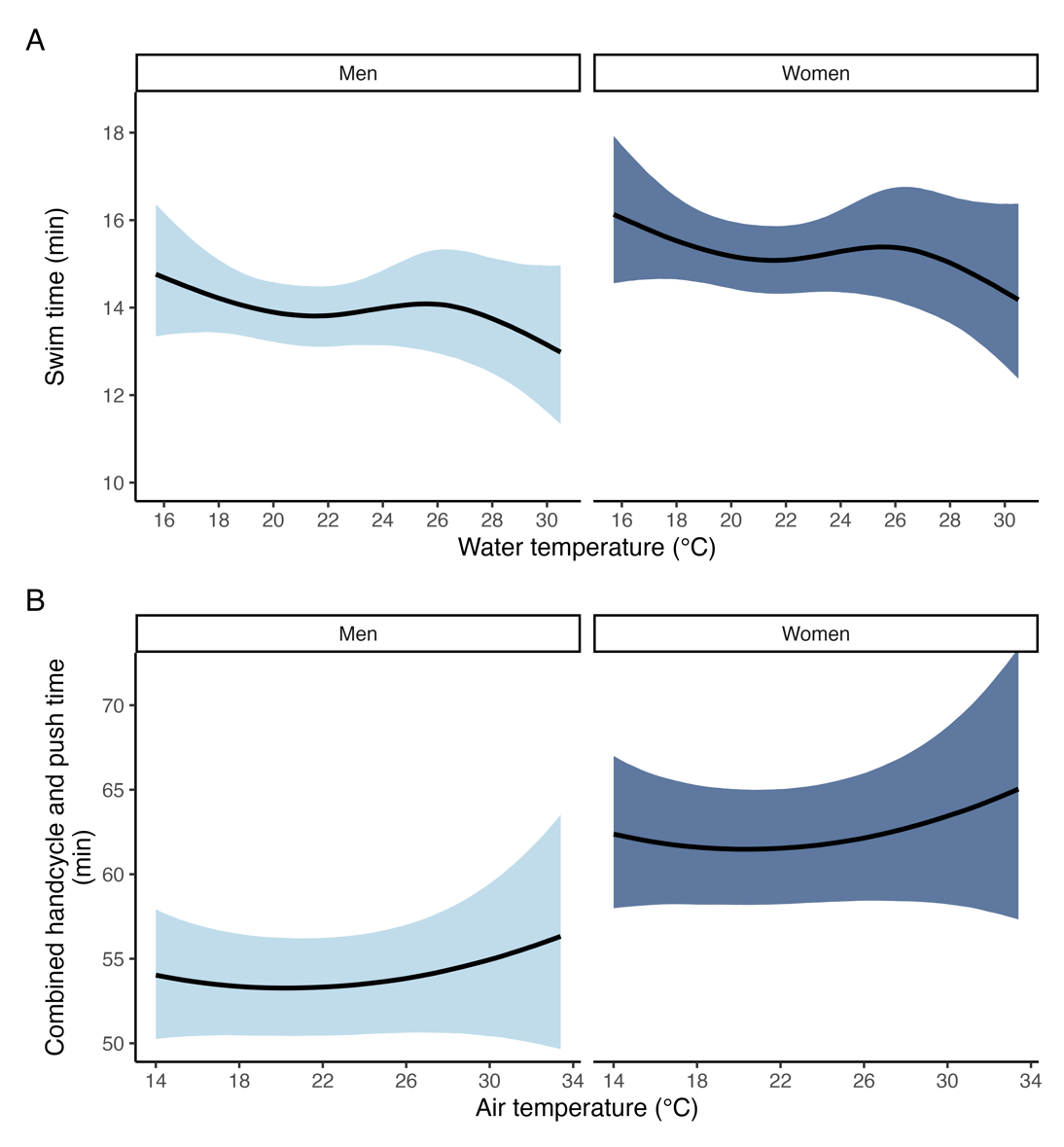


**Supplement 2.** An upset plot showing combinations of missingness across the 575 cases included in the analysis. Missing data was as follows: air temperature 17%, water temperature 14.6%, wet suit information 12.9%, swim time 3.7%, push time 1.7%, handcycle time 1.6% and sport class 0.2%.

**Supplement 3.** Exploratory plots of the relationship between (panel A) water temperature and swim time and (panel B) between air temperature and combined handcycle and push time.


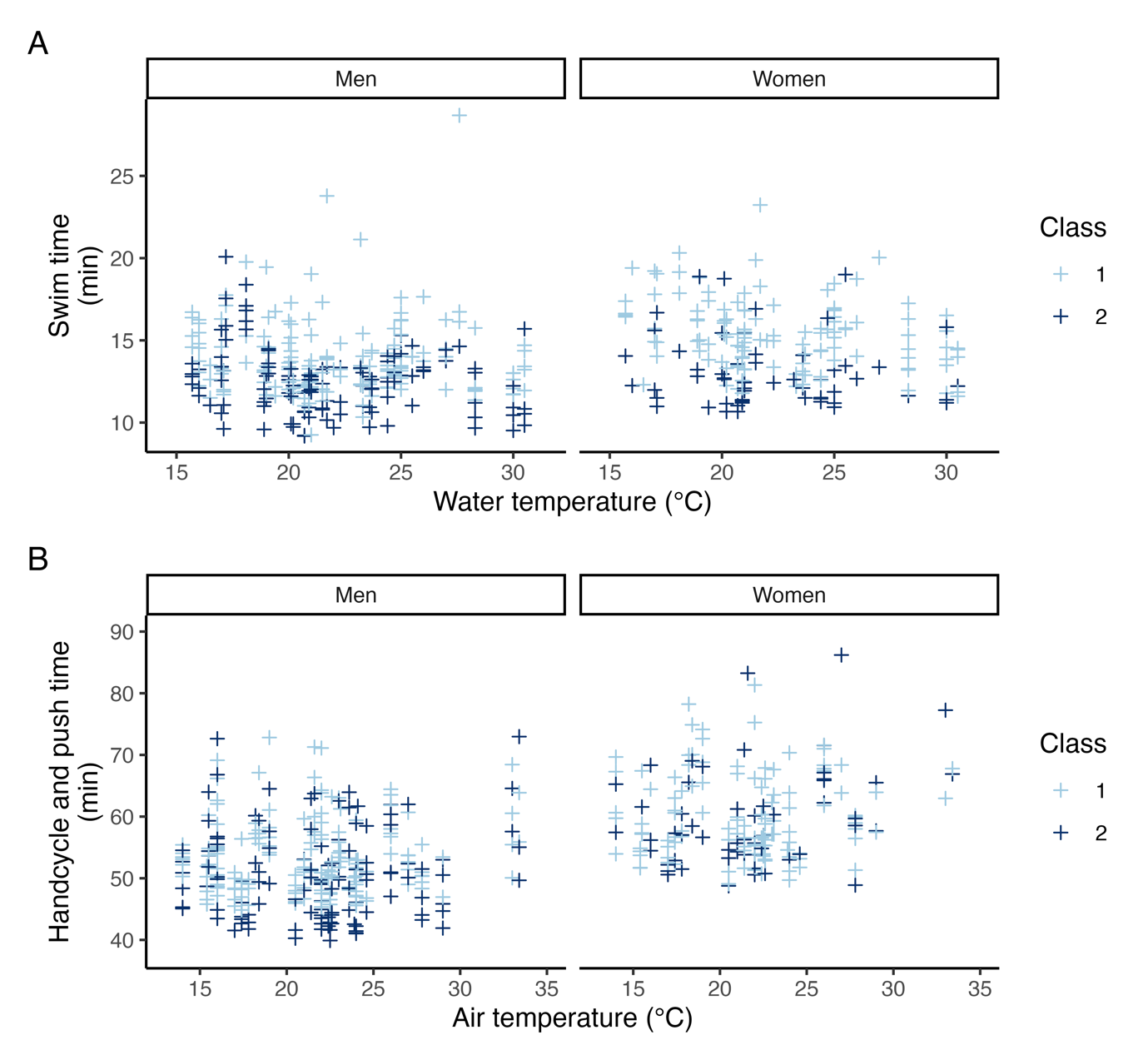


**Supplement 4.** Exploratory plots of the relationship between (panel A) water temperature and swim time and (panel B) between air temperature and combined handcycle and push time, for each sport class. Class 1 athletes have greater impairments than Class 2 athletes. Panel A


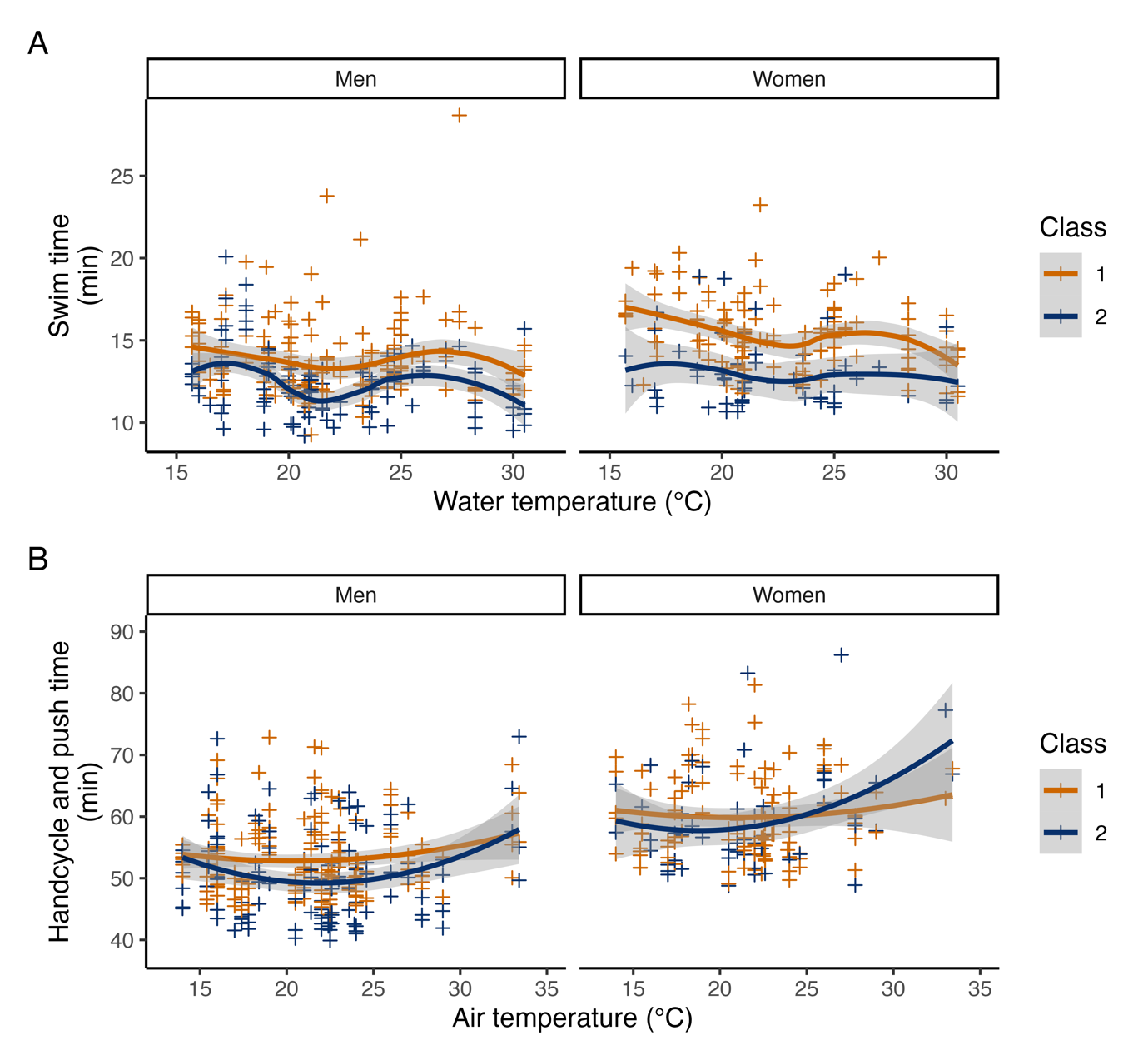

Supplement: Supplemental Material [file KTMP_A_2391170_SM6739.docx]
